# A universal, high-quality, and high-yield DNA purification method for mycobacteria, including *Mycobacterium tuberculosis*: large-scale assessment of the chloroform-bead method

**DOI:** 10.1128/spectrum.00765-25

**Published:** 2025-10-02

**Authors:** Yoshiro Murase, Makiko Hosoya, Yuta Morishige, Yoshiko Shimomura, Miori Nagai, Aki Tamaru, Akiko Takaki, Satoshi Mitarai, on behalf of Japan Tuberculosis Genotyping Group (2023), Yohei Takahashi

**Affiliations:** 1 Aomori Prefectural Institute of Health, Aomori, Japan; 2 Sendai City Institute of Public Health, Sendai, Japan; 3 Kawasaki City Institute for Public Health, Kawasaki, Japan; 4 Sagamihara City Institute of Public Health, Sagamihara, Japan; 5 Aichi Prefectural Institute of Public Health, Nagoya, Japan; 6 Nagoya City Public Health Research Institute, Nagoya, Japan; 7 Kyoto Prefectural Institute of Public Health and Environment, Kyoto, Japan; 8 Osaka Institute of Public Health, Osaka, Japan; 9 Kobe Institute of Health, Kobe, Japan; 10 Himeji City Institute of Environment and Health, Himeji, Japan; 11 Shimane Prefectural Institute of Public Health and Environmental Science, Matsue, Japan; 12 Hiroshima City Institute of Public Health, Hiroshima, Japan; 13 Yamaguchi Prefectural Institute of Public Health and Environment, Yamaguchi, Japan; 14 Fukuoka Institute of Health and Environmental Sciences, Fukuoka, Japan; 15 Nagasaki Prefectural Institute of Environment and Public Health, Nagasaki, Japan; 16 The Oita Prefectural Institute of Health and Environment, Oita, Japan; 1 Department of Mycobacterium Reference and Research, Research Institute of Tuberculosis, Japan Anti-Tuberculosis Association 46635 , Kiyose, Japan; 2 Department of Infectious Diseases, Osaka Institute of Public Health 91397, Osaka, Japan; 3 Department of Basic Mycobacteriosis, Nagasaki University Graduate School of Biomedical Sciences 200674 , Nagasaki, Japan; City of Hope Department of Pathology, Duarte, California, USA

**Keywords:** nontuberculous mycobacteria, genomic analysis, DNA extraction, *Mycobacterium tuberculosis*

## Abstract

Genomic analysis of mycobacteria has become increasingly crucial for understanding drug-resistance mechanisms, molecular epidemiology, and pathogenesis. However, efficient extraction of high-molecular-weight genomic DNA from these organisms remains challenging because of their thick mycolic acid-rich cell walls. In this study, we report the chloroform-bead method, a universal DNA extraction protocol that combines chemical and mechanical disruptions to overcome these challenges. Multi-laboratory evaluation (16 sites) demonstrated the chloroform-bead method’s superiority over conventional methods for *Mycobacterium tuberculosis* (DNA yield: 17.9 vs 1.9 µg, purity A260/A230: 1.86 vs 1.22, both *P* < 0.001). Single-facility assessment extended these findings to >32 nontuberculous mycobacterial species (*n* = 1,058), showing performance comparable to *M. tuberculosis* (*n* = 1,000), with both achieving median yields of 22.2 µg DNA and consistent quality metrics. The chloroform-bead method significantly reduced the processing time from 2 to 3 days to 2 h while ensuring complete sample sterilization, eliminating the need for species-specific optimization. This streamlined and universally applicable protocol represents a practical advancement in mycobacterial DNA extraction methodology, ideal for high-throughput genomic studies and routine clinical diagnostics.

**IMPORTANCE:**

Mycobacterial genomics is crucial for understanding pathogenesis and drug resistance; however, DNA extraction remains a significant challenge because of its unique cell wall. Traditional methods rely on enzymatic treatments, resulting in complex and time-consuming protocols with variable results. The chloroform-bead method introduces a paradigm shift by chemically and mechanically disrupting the mycolic acid layer and eliminating the need for enzymatic treatment. This standardized approach ensures consistent, high-quality DNA extraction across diverse mycobacterial species, thereby enhancing research capabilities and clinical applications.

## INTRODUCTION

Tuberculosis remains a major global public health threat, with 10.8 million new cases and 1.25 million deaths in 2023 (1). Nontuberculous mycobacterial (NTM) infections are increasing worldwide (2). The incidence of NTM infections often surpasses tuberculosis in high-income settings, posing significant challenges because of their intrinsic antibiotic resistance and difficult-to-treat nature. Genomic analysis is crucial for understanding these pathogens and developing enhanced diagnostics, treatments, and prevention strategies (3, 4). However, the unique cell wall structure of mycobacteria complicates the extraction of high-quality genomic DNA for these analyses (5–8).

Conventional DNA extraction methods rely on lysozyme treatment. However, *Mycobacterium tuberculosis* exhibits significant resistance to lysozyme owing to its thick lipid layer in the cell wall (impedes lysozyme penetration), presence of N-glycolylmuramic acid (lysozyme-resistant), and expression of lysozyme-inhibiting lipoproteins (LprI) (9–12).

Current protocols, including cetyltrimethylammonium bromide methods, silica-based techniques, and phenol-chloroform extraction, incorporate additional steps, such as extended lysozyme treatment, freeze-thaw cycles, and proteinase K digestion (5–8). However, these modifications add complexity, increase processing time, and produce inconsistent DNA yield. Additionally, these methods lack universal applicability across diverse mycobacterial species and have been predominantly confirmed in single-facility studies, limiting their broader clinical and research applications.

To address these limitations, we propose the chloroform-bead (CB) method—a novel universal DNA extraction method for mycobacteria. This approach eliminates the need for lysozyme treatment by combining chloroform and bead-beating during initial extraction steps. Chloroform sterilizes bacteria while efficiently removing cell wall lipids, and glass beads mechanically disrupt the cell wall. The method was further simplified by phenol-chloroform purification using phase-lock tubes, enabling straightforward separation of the aqueous layer from organic solvents without specialized expertise. These protocols are designed for universal applicability by reducing the methodological complexity, technical requirements, and processing time.

We have previously reported that DNA obtained using the CB method is sufficient for sequencing the complete genomes of *M. tuberculosis* and NTM species using Illumina, Nanopore, and PacBio HiFi sequencing (13, 14). However, its reproducibility and applicability (crucial factors for global laboratory adoption) across diverse mycobacterial species remain unclear.

In this study, we assessed the feasibility of using the CB method as a universal DNA extraction protocol for mycobacteria. We assessed its performance through a 16-laboratory comparison with conventional methods and large-scale analysis covering 1,058 NTM and 1,000 *M*. *tuberculosis* isolates. This comprehensive validation aimed to establish the CB method as a universal protocol for mycobacterial genomics.

## MATERIALS AND METHODS

### Study design and bacterial isolates

This study used a dual approach to assess the CB method—multi-laboratory assessment for *M. tuberculosis* and large-scale single-facility assessment for NTM species.

For *M. tuberculosis*, four clinical isolates (one each from lineages 1 and 2, and two from lineage 4) were cultured on Ogawa egg-based medium at the Research Institute of Tuberculosis, Japan Anti-Tuberculosis Association (RIT/JATA) and distributed to 16 participating laboratories. Eleven laboratories used the CB method, whereas five used their routine procedures (non-CB methods). The non-CB methods included the following commercial kits: Monarch Genomic DNA Purification Kit (*n* = 2) (New England Biolabs, MA, USA), DNeasy Blood & Tissue Kit (*n* = 2) (QIAGEN, Hilden, Germany), and Maxwell RSC Viral Total Nucleic Acid Purification Kit (*n* = 1) (Promega, WI, USA). Two of these laboratories incorporated bead-beating for 5 min or 2 h before using the kits. The 64 resulting DNA solutions were sent to the RIT/JATA for assessment.

For NTM species, we assessed CB method-processed isolates (1,058) during routine operations from 2022 to 2024 at a single facility, RIT/JATA. The NTM isolates included 602 isolates from the *Mycobacterium abscessus* complex, 164 *Mycobacterium avium*, 119 *Mycobacterium intracellulare*, 50 *Mycobacterium fortuitum*, and 30 *Mycobacterium chelonae*, and 93 isolates from other species, such as *Mycobacterium shinjukuense*, *Mycobacterium peregrinum*, and *Mycobacterium llatzerense* (Table 1). Additionally, for comparison, we included data from 1,000 clinical *M. tuberculosis* isolates processed using the CB method during the same period.

**TABLE 1:** DNA quality and yield from various mycobacterial species using the chloroform-bead method

Species	Number of isolates	A260/A280^*a*^	A260/A230^*a*^	DNA concentration (ng/µL)^*a*^	DNA yield (µg)^*a*^
*M. avium*	165	2.06 (1.95–2.14)	1.95 (1.94–1.97)	238.4 (179.0–285.1)	23.8 (17.9–28.5)
*M. intracellulare*	118	2.13 (1.97–2.18)	1.97 (1.95–1.98)	261.9 (160.0–316.2)	26.2 (16.0–31.6)
*M. abscessus* complex	602	1.93 (1.91–2.09)	1.95 (1.93–2.07)	209.6 (141.1–297.4)	21.0 (14.1–29.7)
*M. chelonae*	30	1.92 (1.89–1.93)	2.08 (2.03–2.12)	189.3 (120.2–344.2)	18.9 (12.0–34.4)
*M. fortuitum*	50	1.92 (1.91–1.93)	2.07 (2.03–2.09)	206.1 (167.2–286.3)	20.6 (16.7–28.6)
Other NTM^*b*^	93	1.93 (1.91–1.96)	1.98 (1.93–2.08)	261.7 (183.3–374.2)	26.2 (18.3–37.4)
All NTM	1,058	1.94 (1.91–2.11)	1.96 (1.94–2.05)	221.9 (151.5–307.4)	22.2 (15.1–30.7)
*M. tuberculosis*	1,000	1.92 (1.91–1.94)	1.91 (1.75–2.03)	222.0 (139.2–337.4)	22.2 (13.9–33.7)

^*a*^ Values are presented as median (interquartile range).

^*b*^ Other NTM include *M. shinjukuense* (*n* = 14), *M. peregrinum* (*n* = 14), *M. llatzerense* (*n* = 7), *Mycobacterium kiyosense* (*n* = 6), *Mycobacterium florentinum* (*n* = 6), *Mycobacterium mucogenicum* (*n* = 4), and 21 additional species with ≤3 isolates each plus suspected novel species (*n* = 9).

### Chloroform-bead DNA extraction for mycobacteria

The step-by-step protocol is provided in the Supplemental material. One loopful of mycobacterial cells (approximately 10 mg) from solid media was added to 2.0 mL screw-cap tubes containing 600 mg of 0.2 mm diameter glass beads (AZ ONE Co. Ltd., Osaka, Japan), 700 µL of 0.1 M NaCl/TE buffer (10 mM Tris-HCl, 1 mM EDTA, pH 8.0), and 500 µL of chloroform. The cells were disrupted by vortexing at 2,700 rpm for 7 min using a VORTEX-GENIE 2 with Turbomix Attachment (Scientific Industries Inc., NY, USA). The resulting mixture was treated with RNase A for 20 min, followed by phenol-chloroform and chloroform extractions in a phase-lock tube (QIAGEN, Hilden, Germany). Finally, the DNA was precipitated using isopropanol and dissolved in 100 µL elution buffer (10 mM Tris-HCl, pH 8.5).

### Sterilization effect of the chloroform-bead method for mycobacteria

To assess the sterilization efficacy of the CB method, we processed 100 clinical *M. tuberculosis* isolates using a standard CB protocol. Following CB disruption and centrifugation, we collected the supernatant and interphase layers and inoculated them into liquid (mycobacteria growth indicator tube) and solid media (7H10 or Ogawa). The cultures were monitored for bacterial growth for 12 weeks.

### DNA quality assessment

Purified DNA was quantified using NanoDrop spectrophotometer and Qubit fluorometer (Thermo Fisher Scientific Inc., Waltham, MA, USA). DNA purity was assessed using A260/A280 and A260/A230 ratios. The Qubit/NanoDrop ratio was used to assess the proportion of double-stranded DNA, with values closer to 1 indicating higher purity. DNA molecular weight distribution was analyzed using the TapeStation 4200 system (Agilent Technologies, CA, USA), where the DNA integrity number (DIN) and top peak values were measured.

### Statistical analysis

After assessing data normality using the Shapiro-Wilk test, we used non-parametric methods for comparisons. The Mann-Whitney U-test was used to compare the CB and non-CB methods, and effect sizes were calculated using Cliff’s delta. Deviation of the Qubit/NanoDrop ratio from 1 was assessed using the Wilcoxon signed-rank test. To control false positives in multiple comparisons, *P*-values were adjusted using the false discovery rate method. Results were summarized using median and interquartile range (IQR) and visualized with box plots. Statistical analyses were performed using R version 4.3.2, with a 5% significance level. Cliff’s delta values were interpreted as follows: <0.147 negligible, <0.33 small, <0.474 medium, and ≥0.474 large effect (15).

### Scanning electron microscopy (SEM) observation

To assess the mechanism of DNA extraction using the CB method, SEM was performed on the interphase layer obtained during the CB treatment of *M. tuberculosis* H37Rv. Untreated H37Rv colonies grown on Löwenstein-Jensen medium were used as negative controls. The samples were fixed with 2.5% glutaraldehyde in phosphate buffer (4°C, 24 h), washed with phosphate buffer, and then fixed with 1% osmium tetroxide (4°C, 1 h). Following dehydration using a graded ethanol series and tert-butyl alcohol substitution, the samples were freeze-dried overnight using a JFD-300 freeze dryer (JEOL, Tokyo, Japan). The dried specimens were mounted on brass stubs, coated with a 10 nm gold layer using a Q150T ES ion sputtering device (Quorum Technologies, East Sussex, UK), and observed using a scanning electron microscope (IT-800SHL; JEOL, Tokyo, Japan).

## RESULTS

### Comparison of the CB method with non-CB methods (multi-laboratory assessment)

#### DNA purity comparison

**Fig 1:**
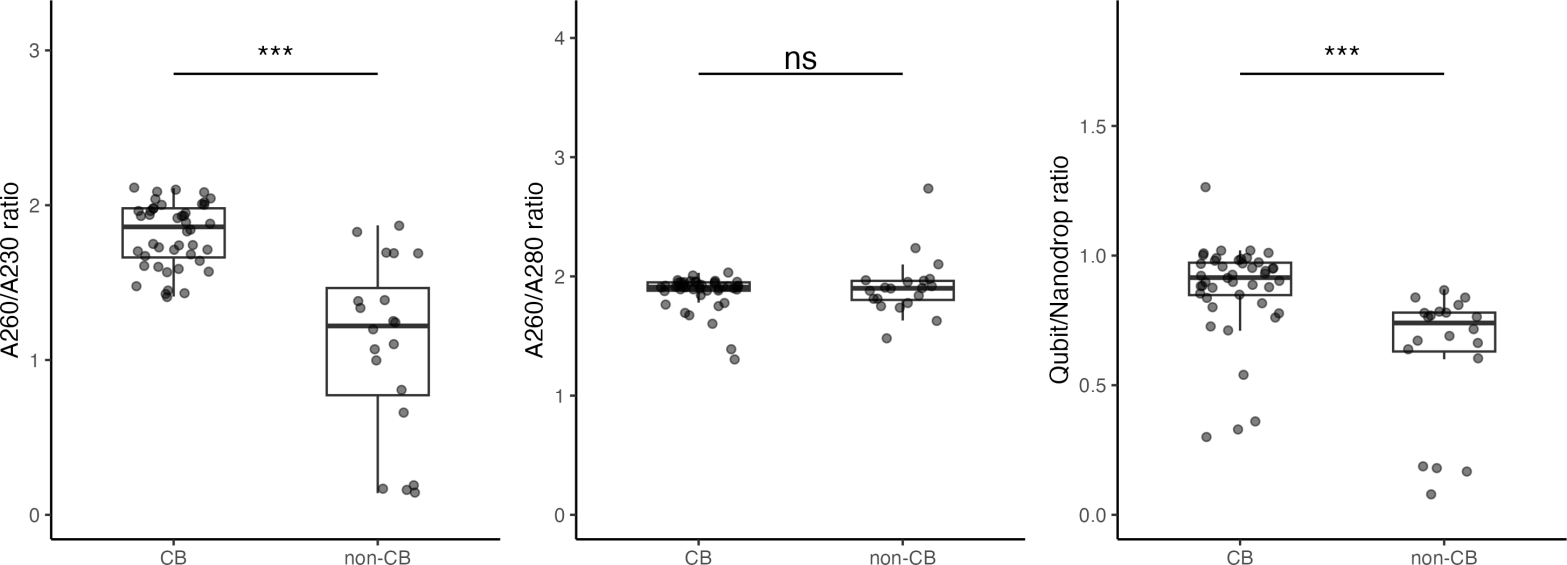
Multi-laboratory assessment of DNA quality: CB disruption method vs conventional approaches. Comparative analysis of DNA quality extracted from four clinical *Mycobacterium tuberculosis* isolates across 16 laboratories using the CB disruption method (11 laboratories, *n* = 44) and non-CB methods (5 laboratories, *n* = 20). (Left panel) A260/A230 ratio, indicating organic contamination. (Center panel) A260/A280 ratio, indicating protein contamination. (Right panel) Qubit/NanoDrop ratio, a measure of quantification accuracy (optimal value: 1.0). Box plots: interquartile range; points: individual samples. *P < 0.05, **P < 0.01, and ***P < 0.001 (Mann-Whitney U-test, false discovery rate-corrected). Effect sizes (Cliff’s delta, δ) between CB and non-CB groups: (Left panel) δ = 0.81, (Center panel) δ = 0.03, and (Right panel)δ = 0.75. Interpretation: small |δ| < 0.33, medium 0.33 ≤ |δ| <0.47, and large |δ| ≥ 0.47.

The CB method produced DNA with superior purity, characterized by reduced organic contamination and higher double-stranded DNA specificity (Fig. 1). The A260/A280 ratio exhibited no significant difference between the methods (median: CB 1.91 vs non-CB 1.90, *P* = 0.866), indicating similar protein contamination levels. The A260/A230 ratio was significantly higher in the CB method (median: 1.86 vs 1.22, *P* < 0.001), with a large effect size (Cliff’s delta = 0.81), indicating reduced organic contamination. The Qubit/NanoDrop ratio that specifically reflects the double-stranded DNA content was significantly higher in the CB method (median: 0.92 vs 0.74, *P* < 0.001, Cliff’s delta = 0.75), indicating reduced contamination by RNA, single-stranded DNA, and other UV-absorbing impurities that can inflate NanoDrop readings.

#### DNA yield comparison

**Fig 2:**
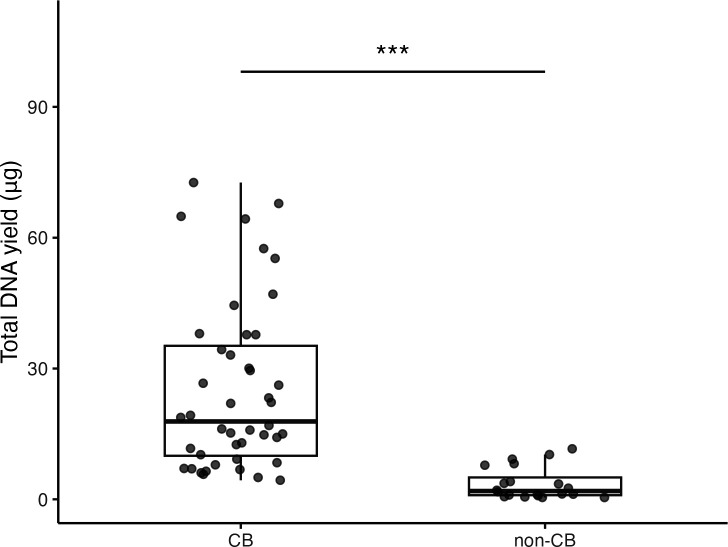
Multi-laboratory assessment of DNA yield: CB disruption method vs conventional methods. Assessment of total DNA yield (μg) from four clinical *Mycobacterium tuberculosis* isolates across 16 laboratories, comparing the CB method (11 laboratories, *n* = 44) and non-CB methods (5 laboratories, *n* = 20). Non-CB methods include bead disruption using silica/magnetic bead purification (three laboratories) and silica column purification (two laboratories). Box plots: interquartile range; points: individual samples. ***P < 0.001 (Mann-Whitney U-test, false discovery rate-corrected). Effect sizes (Cliff’s delta, δ) between CB and non-CB groups: (Left) δ = 0.88, (Right) δ = 0.86. Interpretation: small |δ| < 0.33, medium 0.33 ≤ |δ| < 0.47, and large |δ| ≥ 0.47.

The CB method produced significantly higher DNA yields compared to that of non-CB methods (median: 17.9 µg vs 1.9 µg, *P* < 0.001) (Fig. 2). Data from 16 laboratories demonstrated that the CB method (*n* = 44) consistently outperformed non-CB methods (*n* = 20), with approximately 9.4-fold higher yields. The effect size (Cliff’s delta = 0.88) indicated a significant practical difference between the methods. Although the CB method exhibited higher variability in absolute yields (IQR: 15.2 µg–21.4 µg) than that of non-CB methods (IQR: 0.8 µg–3.2 µg), it consistently produced sufficient DNA for downstream applications, including whole-genome sequencing.

#### DNA molecular weight comparison

**Fig 3:**
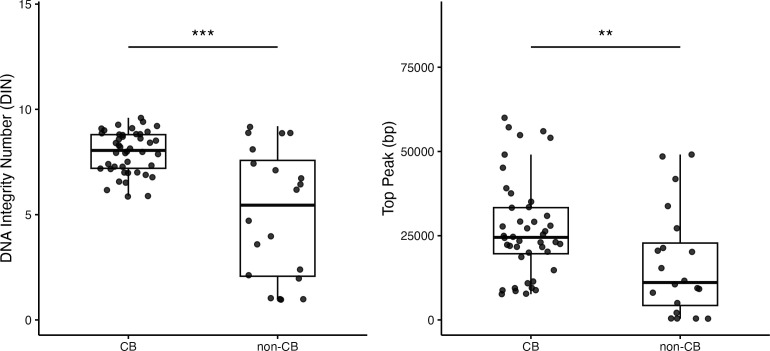
Multi-laboratory assessment of DNA integrity and top peak size: CB disruption method vs conventional methods. Analysis of DNA integrity from four clinical *Mycobacterium tuberculosis* isolate extractions across 16 laboratories, comparing the CB method (11 laboratories, *n* = 44) and non-CB methods (5 laboratories, *n* = 20). (Left panel) DNA integrity number, indicating overall DNA quality. (Right panel) Top peak values (bp) represent the most prevalent DNA fragment length. Box plots: interquartile range; points: individual samples. *P < 0.05, **P < 0.01 and ***P < 0.001 (Mann-Whitney U-test, false discovery rate-corrected). Effect sizes (Cliff’s delta, δ) between CB and non-CB groups: (Left panel) δ = 0.53, (Right panel) δ = 0.43. Interpretation: small |δ| < 0.33, medium 0.33 ≤ |δ| < 0.47, and large |δ| ≥ 0.47.

The CB method produced DNA with higher molecular weight and integrity than that of non-CB methods (Fig. 3). The DIN was significantly higher for the CB method (median: 8.05 vs 5.45, *P* < 0.001) with a large effect size (Cliff’s delta = 0.53). Similarly, the top peak size was significantly larger for the CB method (median: 24,513 vs 11,105 bp, *P* < 0.01), with a medium effect size (Cliff’s delta = 0.43). The CB method demonstrated more consistent DIN values across samples, indicating reliable extraction of high-integrity DNA suitable for long-read sequencing applications.

### Assessment of DNA purification from NTM species (single-facility assessment)

The CB method, originally developed for *M. tuberculosis*, was assessed for its ability to extract DNA from NTM species (Table 1). Quantitative analysis revealed that DNA yields were comparable between NTM (median: 22.2 µg, IQR: 15.1 µg–30.7 µg) and *M. tuberculosis* (median: 22.2 µg, IQR: 13.9 µg–33.7 µg) isolates. Additionally, DNA purity was consistently high in both groups. The A260/A280 ratios for NTM and *M. tuberculosis* were 1.94 (IQR: 1.91–2.11) and 1.92 (IQR: 1.91–1.94), respectively, whereas the A260/A230 ratios were 1.96 (IQR: 1.94–2.05) and 1.91 (IQR: 1.75–2.03), respectively. These values aligned with or slightly exceeded the expected range for pure DNA (A260/A280: approximately 1.8; A260/A230: 1.8–2.2) (16), indicating that the CB method consistently produces DNA of acceptable purity for downstream applications.

A detailed analysis of major NTM species, such as *M. avium*, *M. intracellulare*, and the *M. abscessus* complex, demonstrated consistently high DNA yields (median ≥18.9 µg) and purity (median A260/A280 ≥ 1.92; median A260/A230 ≥ 1.95), with relatively minor interspecies variations. These results confirmed the broad applicability of the CB method to diverse mycobacterial species.

### Safety confirmation of the CB method

The sterilization efficacy of the CB method was assessed using 100 clinical isolates of *M. tuberculosis*. After CB disruption, the supernatant and interphase layers were cultured in liquid and solid media for 12 weeks. No bacterial growth was observed in any of the processed samples (0/100), whereas all positive controls (untreated *M. tuberculosis* culture) demonstrated growth (3/3) (Table 2). These results demonstrated that the CB method completely sterilizes *M. tuberculosis*, confirming its safety for laboratory use.

**TABLE 2:** *Mycobacterium tuberculosis* viability assessment after CB disruption

Sample type	Number of samples	Liquid media^*a*^	Solid media^*b*^	Incubation period (weeks)
Supernatant	100	0/100	0/100	12
Interphase layer	100	0/100	0/100	12
Positive control^*c*^	3	3/3	3/3	12
Negative control^*d*^	3	0/3	0/3	12

^*a*^ Mycobacteria growth indicator tube is used as liquid media.

^*b*^ 7H10 and Ogawa media are used as solid media; both are confirmed for *M. tuberculosis* culture.

^*c*^ *M. tuberculosis* without CB treatment.

^*d*^ CB DNA extraction buffer without *M. tuberculosis* inoculation.

### Morphological analysis of CB-treated cells

SEM revealed distinct morphological alterations in *M. tuberculosis* cells following CB treatment. The untreated cells exhibited a characteristic rod-shaped morphology with well-defined boundaries (Fig. S1a and b). In contrast, the CB-treated cells were coated with water-insoluble structures (Fig. S1c and d), indicating the extraction and accumulation of cellular components on the surface.

## DISCUSSION

This study assessed the CB method for mycobacterial DNA extraction using two complementary approaches—multi-laboratory assessment (*n* = 16) focusing on *M. tuberculosis* and large-scale single-facility assessment covering both *M. tuberculosis* (*n* = 1,000) and >32 NTM species (*n* = 1,058). In the multi-laboratory assessment, the CB method consistently outperformed conventional methods in DNA yield (median 17.9 vs 1.9 µg, *P* < 0.001), purity (A260/A230: 1.86 vs 1.22, *P* < 0.001), and molecular weight (top peak size: 24,513 vs 11,105 bp, *P* < 0.01) (Fig. 1 to 3). The single-facility assessment confirmed these advantages across diverse mycobacterial species, demonstrating universal applicability (Table 1).

The CB method offers three major advantages. First, it reduces processing time from 2 to 3 days to approximately 2 h by eliminating lysozyme treatment and freeze-thaw cycles. Second, it provides a universal protocol without species-specific optimization, demonstrated by consistent performance even with challenging species like *M. abscessus*, where conventional methods showed three orders of magnitude of variability between isolates (8). Third, the multi-laboratory assessment confirmed robust inter-laboratory reproducibility, essential for protocol standardization.

A recent study in 2024 (17) has also highlighted the importance of mechanical lysis in mycobacterial DNA extraction. While their approach validates the utility of bead-beating from liquid mycobacteria growth indicator tube (MGIT; Becton Dickinson) cultures of *M. tuberculosis* in a single-facility setting, our study further extends its application to a broader range of mycobacterial species and demonstrates the robustness of our methodology through a multi-facility-based assessment. We demonstrated that the CB method can extract sufficient DNA (1.3 µg [IQR: 0.85–1.6, *n* = 20]) from MGIT tubes on the same day they turn positive, with 100% success rate of routine Illumina sequencing, yielding ≥86% Q30 scores and read depths ≥64× when mapped to H37Rv (data not shown), enabling early application of genomic data for patient treatment and outbreak control. Furthermore, the CB method produces longer DNA fragments (N50: 17.1 kb [IQR: 9.8–18.1, *n* = 28]) (13, 14) compared to bead-beating alone (N50: 1.4 kb–2.6 kb) (17), which is particularly valuable for determination of complete genome with long-read sequencing.

The effectiveness of the CB method stems from the combined action of chemical and mechanical cell disruptions. Electron microscopy revealed that the CB-treated cells were coated with water-insoluble structures (Fig. S1), indicating that chloroform-mediated lipid removal and bead-beating effectively extracted cellular components. This less severe extraction process likely contributes to the high molecular weight of the extracted DNA (Fig. 3).

The biosafety of the CB method was confirmed by complete inactivation of *M. tuberculosis* in the processed samples (Table 2). This is a crucial feature for routine clinical use because it minimizes the risk of laboratory-acquired infections.

Despite these advantages, the CB method has limitations. First, the use of chloroform and phenol poses environmental and safety concerns, necessitating future development of safer alternatives. Second, our sterilization validation was performed after bead-beating in chloroform-containing tubes (Supplemental material). Laboratories requiring pre-vortexing sterilization should independently validate chloroform-immersion-alone effectiveness. Third, while we demonstrated consistent performance across >32 NTM species (*n* = 1,058), we did not perform the multi-laboratory validation, limiting conclusions about inter-laboratory reproducibility for NTM. Furthermore, we did not compare the CB and conventional methods for NTM.

In conclusion, the CB method advances mycobacterial DNA extraction, providing a rapid, universal approach with confirmed biosafety. The CB method will facilitate large-scale genomic studies on *M. tuberculosis* and NTM infections, supporting both basic research and clinical applications. Its robust performance and confirmed protocol establish a foundation for future developments in mycobacterial genomics and precision medicine.

## SUPPLEMENTAL MATERIAL

Fig. S1 spectrum.00765-25-s0001.pdf Scanning electron microscopy of *Mycobacterium tuberculosis* H37Rv cells before and after chloroform-bead treatment.

Supplemental material spectrum.00765-25-s0002.pdf Protocol for chloroform-bead DNA extraction method.

ASM does not own the copyrights to Supplemental Material that may be linked to, or accessed through, an article. The authors have granted ASM a non-exclusive, world-wide license to publish the Supplemental Material files. Please contact the corresponding author directly for reuse.
