# Supplementary material for: A universal, high-quality, and high-yield DNA purification method for mycobacteria, including Mycobacterium tuberculosis: large-scale assessment of the chloroform-bead method
Source: Microbiol Spectr. 2025 Oct 2;13(11):e00765-25. doi: 10.1128/spectrum.00765-25 (PMC12584714; doi:10.1128/spectrum.00765-25)
Supplement: Fig. S1 — Scanning electron microscopy of Mycobacterium tuberculosis H37Rv cells before and after chloroform-bead treatment. [file spectrum.00765-25-s0001.pdf]

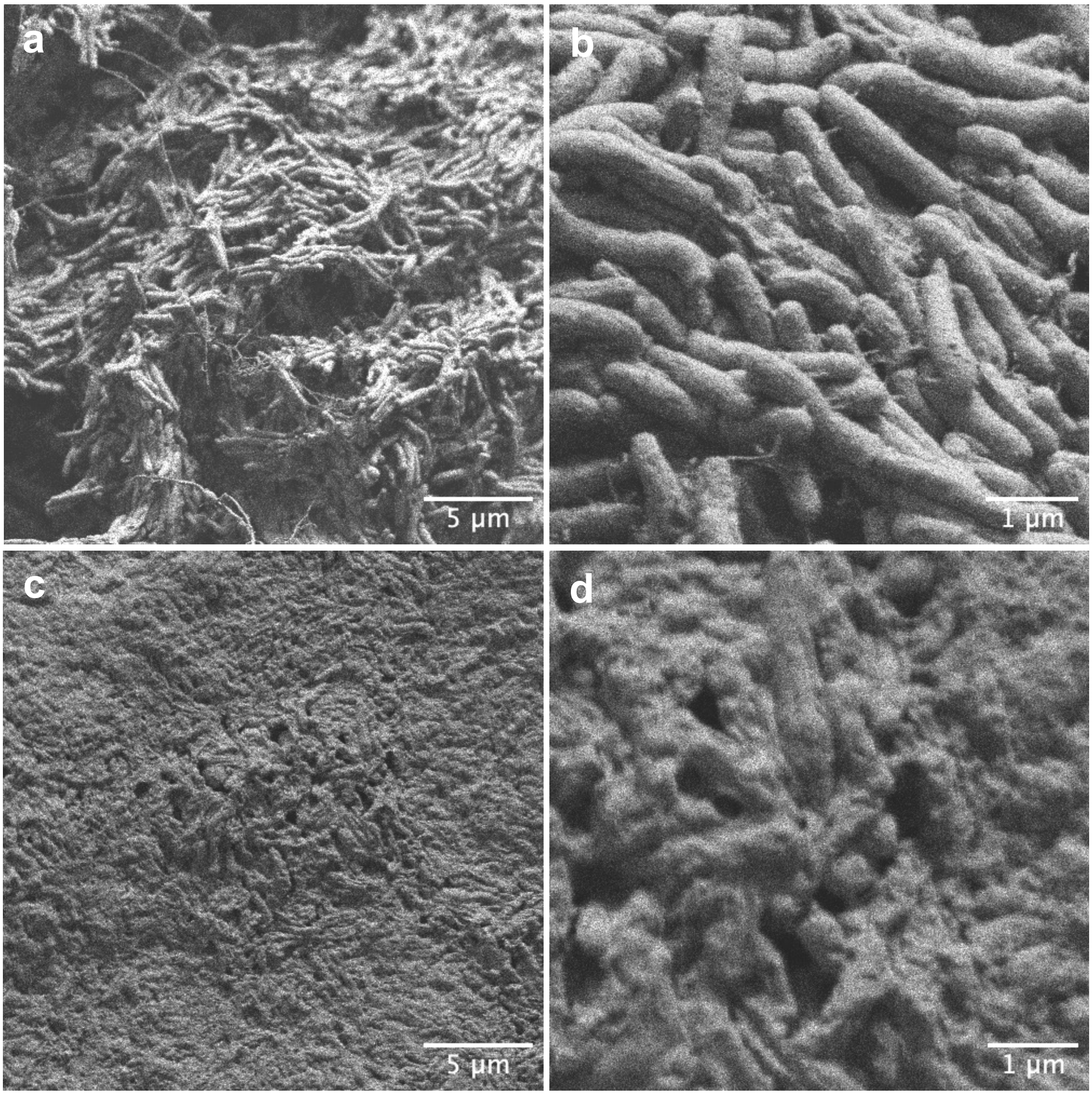

Supplemental Figure. Scanning electron microscopy of *Mycobacterium tuberculosis* H37Rv cells before and after chloroform-bead (CB) treatment.

(a, b) Untreated cells cultured on a solid medium demonstrating typical rod-shaped morphology at low (a, scale bar: 5 μm) and high (b, scale bar: 1 μm) magnification. (c, d) CB-treated cells coated with water-insoluble structures at low (c, scale bar: 5 μm) and high (d, scale bar: 1 μm) magnification. Images were acquired using low-angle backscattered electron imaging.
