# Supplementary material for: A universal, high-quality, and high-yield DNA purification method for mycobacteria, including Mycobacterium tuberculosis: large-scale assessment of the chloroform-bead method
Source: Microbiol Spectr. 2025 Oct 2;13(11):e00765-25. doi: 10.1128/spectrum.00765-25 (PMC12584714; doi:10.1128/spectrum.00765-25)
Supplement: Supplemental material — Protocol for chloroform-bead DNA extraction method. [file spectrum.00765-25-s0002.pdf]

Supplemental Protocol

A. Chloroform-bead DNA extraction protocol

BSL3 Laboratory Steps

1. Transfer one loopful (approximately 10 mg) of Mycobacterium pellet to the DNA extraction tube
2. Vortex at 2700 rpm for 7 min using Vortex Mixer GENIE2 equipped with Turbomix Attachment (Scientific Industries Inc., NY, USA)

Biosafety Note: Steps 1 and 2 must be performed within a certified Class II Biosafety Cabinet to minimize the risk of aerosol generation.

Standard Laboratory Steps

3. Centrifuge at 10,000 rpm for 10 min using an angle rotor
4. Transfer 600 µL of the supernatant to a Phase lock tube
5. Add 50 µL of RNase A (0.65 µg/µL) and incubate at 37 °C for 20 min
6. Add 600 µL of phenol:chloroform:isoamyl alcohol (PCIAA), mix vigorously until emulsified, and centrifuge at 3,500 rpm for 5 min
7. Repeat step 6
8. Add 600 µL of chloroform, mix vigorously, and centrifuge at 3,500 rpm for 10 min
9. Transfer 500 µL of the supernatant to a 1.5 mL tube
10. Add 50 µL of 3 M NaOAc and 500 µL of isopropyl alcohol (IPA) and mix well
11. Centrifuge at 15,000 rpm for 15 min
12. Discard the supernatant and rinse the DNA pellet with 1 mL of 75 % ethanol
13. Lightly centrifuge and remove any residual ethanol
14. Air dry the DNA pellet for 60 s
15. Add 100 µL of elution buffer (EB) to dissolve the DNA

Total processing time: Approximately 2 h including 10 min in BSL3

Note:

1. For applications requiring high molecular weight DNA (long-read sequencing) or if DNA purity is insufficient:
   - Purify the DNA using 0.7× AMPure XP beads (Beckman Coulter Life Sciences, Indianapolis, USA) following the manufacturer's protocol after Step 15.
   - This step removes low molecular weight DNA fragments
2. For further enrichment of high molecular weight DNA, reduce the vortex time in Step 2 from 7 min to 3 min to minimize DNA fragmentation

B. Preparation

- DNA extraction tube: Prepare a 2 mL screw-cap tube with 600 mg of 0.2 mm glass beads and 700 μL of 0.1 M NaCl/TE (10:1, pH 8.0). Add 500 μL of chloroform on the day of use.
- Phase lock tube:
  - Commercial tubes: MaXtract High Density (15 mL, QIAGEN)
  - Self-made tubes: Use a 10–15 mL centrifuge tube (V-bottom with screw cap). Add 3 g of high-vacuum silicone grease (HVG-50 [Toray Dow Corning, Tokyo, Japan]) and centrifuge lightly.
- RNase A: 0.65 μg/μL
- PCIAA (25:24:1)
- Chloroform
- 3 M NaOAc
- Isopropyl Alcohol
- 75 % EtOH
- EB (10 mM Tris-HCl, pH 8.5, QIAGEN)

C. Appendixes figures: primary steps in the chloroform-bead (CB) extraction method

This appendix provides photographic documentation of primary steps in the CB extraction method, highlighting primary points to aid in the understanding and execution of the protocol.

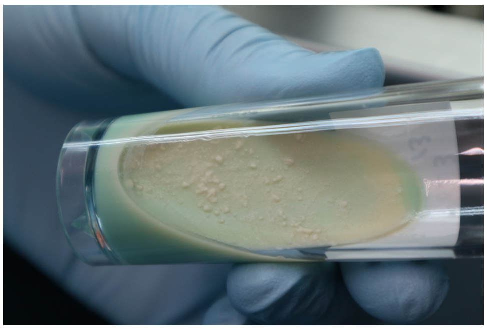

Step 1a: *Mycobacterium tuberculosis* on solid medium.

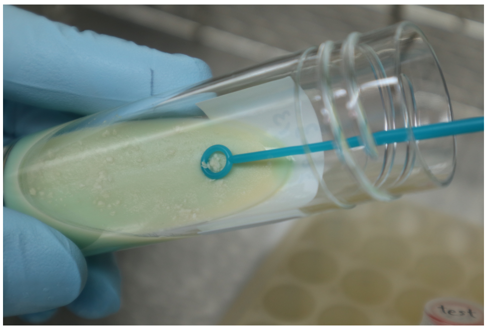

Step 1b: Use an inoculation loop to collect the bacterial pellet.

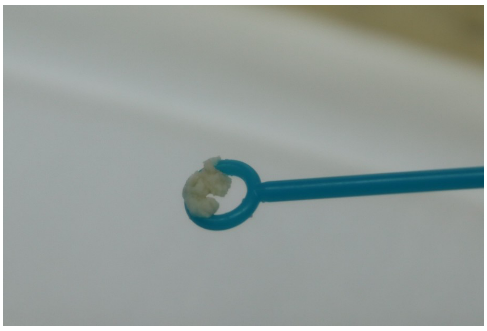

Step 1c: Use approximately 0.5–1 loop of bacterial pellet (approximately 10 mg).

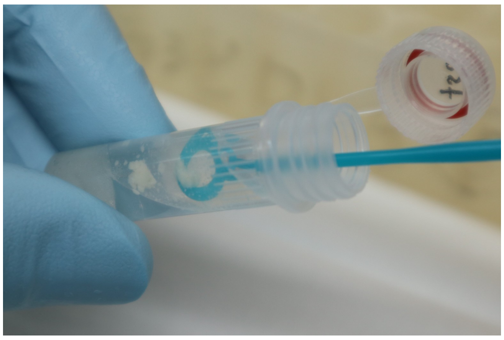

Step 1d: Transfer the bacterial pellet to the DNA extraction tube.

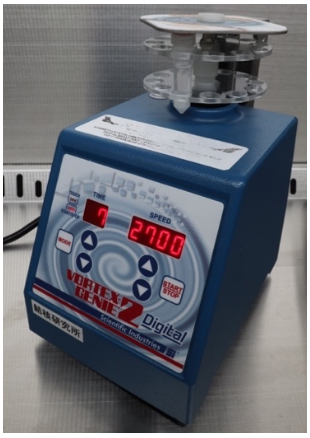

Step 2: Vortex the DNA extraction tube containing glass beads to extract DNA from the bacterial cells on Vortex Mixer GENIE2 with Microtube Attachment at maximum speed for 7 min.

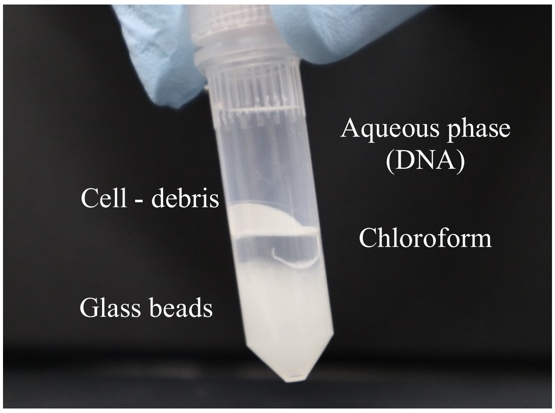

Step 3: Centrifuge the tube and recover the aqueous phase containing DNA.

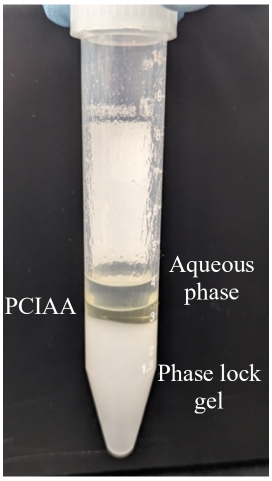

Step 6a: Add PCIAA to the Phase Lock Gel tube containing the aqueous phase.

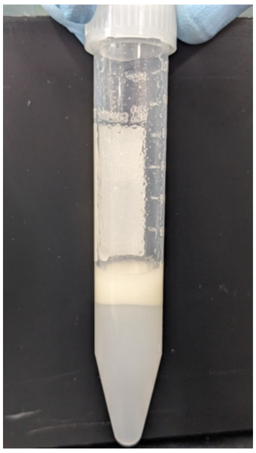

Step 6b: Vigorously mix the aqueous phase and organic solvent by shaking approximately 50 times to purify DNA.

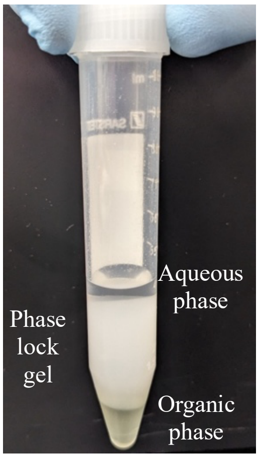

Step 6c: Centrifuge to separate the aqueous phase and organic solvent. Repeat the organic solvent extraction steps in the same tube.

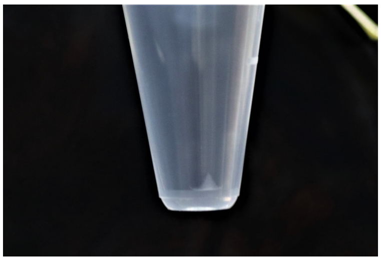

Step 11: The recovered DNA pellet.
